# Expression of SFRP Family Proteins in Human Keratoconus Corneas

**DOI:** 10.1371/journal.pone.0066770

**Published:** 2013-06-18

**Authors:** Jingjing You, Li Wen, Athena Roufas, Michele C. Madigan, Gerard Sutton

**Affiliations:** 1 Save Sight Institute & Discipline of Clinical Ophthalmology, University of Sydney, Sydney, New South Wales, Australia; 2 School of Optometry & Vision Sciences, University of New South Wales, Kensington, New South Wales, Australia; 3 Auckland University, Auckland, New Zealand; 4 Vision Eye Institute, Chatswood, New South Wales, Australia; University of Illinois at Chicago, United States of America

## Abstract

We investigated the expression of the secreted frizzled-related proteins (SFRPs) in keratoconus (KC) and control corneas. KC buttons (∼8 mm diameter) (n = 15) and whole control corneas (n = 7) were fixed in 10% formalin or 2% paraformaldehyde and subsequently paraffin embedded and sectioned. Sections for histopathology were stained with hematoxylin and eosin, or Periodic Acid Schiff’s reagent. A series of sections was also immunolabelled with SFRP 1 to 5 antibodies, visualised using immunofluorescence, and examined with a Zeiss LSM700 scanning laser confocal microscope. Semi-quantitative grading was used to compare SFRP immunostaining in KC and control corneas. Overall, KC corneas showed increased immunostaining for SFRP1 to 5, compared to controls. Corneal epithelium in all KC corneas displayed heterogeneous moderate to strong immunoreactivity for SFRP1 to 4, particularly in the basal epithelium adjacent to cone area. SFRP3 and 5 were localised to epithelial cell membranes in KC and control corneas, with increased SFRP3 cytoplasmic expression observed in KC. Strong stromal expression of SFRP5, including extracellular matrix, was seen in both KC and control corneas. In control corneas we observed differential expression of SFRP family proteins in the limbus compared to more central cornea. Taken together, our results support a role for SFRPs in maintaining a healthy cornea and in the pathogenesis of epithelial and anterior stromal disruption observed in KC.

## Introduction

The cornea is important for protection of the eye and is essential for vision. The central cornea is a key component for transmitting light to the retina, and provides approximately two thirds of the total refractive power of the human eye [1]. The cornea comprises an outer non-keratinised epithelium, Bowman’s layer, stroma, Descemet’s membrane and endothelium. In the periphery, the cornea transitions to the limbus, a narrow zone that separates the cornea from the conjunctiva and underlying sclera. The limbus contains stem cell niches within the basal epithelial papillae of the Palisades of Vogt that are critical for repopulating the corneal epithelial cells, and also act as a barrier to the ingrowth of the conjunctiva and blood vessels [2].

Keratoconus (KC) is a bilateral progressive, asymmetric, degenerative anterior corneal disease (ectasia) that usually presents in the 2nd decade and progresses into the 3rd and 4th decade [3]. KC is associated with decreasing visual function related to progressive corneal thinning and development of irregular astigmatism and myopia [4]. Epithelial basement membrane irregularities and thinning, development of a conical corneal shape, remodelling and loss of corneal nerves, anterior stromal thinning and keratocyte apoptosis are considered characteristic features of KC pathogenesis [5]–[7]. Although the aetiology of KC is still unclear, the evidence from many studies suggests that both genetic and environmental factors are involved [3], [8]. Genes including VSX1, ZEB1, SOD1, TGFB1, MIR184, COL4A3/COL4A4, RAB3GAP1, LOX, HGF and DOCK9 are reported to be associated with KC [8], and atopy and eye rubbing are considered the two main environmental factors linked to KC [3], [9].

We recently reported significantly increased SFRP1 mRNA in KC epithelium compared to control corneal epithelium, suggesting its potential involvement in the pathogenesis of KC [10]. Iqbal et al. (2013) recently confirmed that SFRP1 protein expression is significantly increased in KC corneas compared to control and Fuch’s dystrophy corneas [11]. SFRP1 belongs to the secreted glycoprotein SFRP family (SFRP1 to 5), which are antagonists of Wnt signalling pathways [12]. The Wnt signalling pathways, including both canonical (Wnt/β-catenin) and non-canonical (Wnt/Ca^2+^ and planar-cell-polarity (PCP)) pathways, are a complex network of proteins involved in controlling many physiological processes in mammals including cell proliferation, cell migration and differentiation [13], and regulation of inflammation [14]. These pathways play a critical role in the normal development of the vertebrate eye [15].

Currently little is known about the expression of SFRPs in adult human cornea. The Wnt canonical pathway has been reported to regulate the proliferation of adult human corneal limbal stem cells [16]. However, this study primarily investigated the expression of Wnt molecules, and mRNAs of only two SFRPs (SFRP3 and 5) [16]. Exogenous SFRP1 has been reported to delay corneal epithelial wound healing [17], and also protect corneal epithelial cells against benzalkonium chloride toxicity [18]. These recent studies together with our earlier findings in KC, suggest a role for SFRPs in normal corneal function and in corneal disease. In this study, we performed a systematic study of SFRP1 to 5 expression and distribution in human control corneas and KC buttons. We also examined for SFRPs in the control corneal periphery and limbus, a known stem-cell niche important for corneal epithelial renewal [2], [19].

## Materials and Methods

### Ethics Statement

Ethics approval was obtained from the Sydney Eye Hospital Human Research Ethics Committee (HREC Ref 07/088), and all procedures were in accordance with the Declaration of Helsinki. Written informed consent was obtained from all participants prior to collection of keratoconus buttons. Normal donor corneas were obtained by the Lions NSW Eye Bank with written and verbal recorded consent and Human Research Ethics Committee approval.

### Corneal Specimens

Fifteen corneal buttons were collected from KC patients (age range 21 to 58 years) undergoing corneal transplantation at Vision Group, Chatswood, NSW, Australia. All KC patients had been previously diagnosed on the basis of clinical signs and corneal topography and were classified as KC grade 4 (most severe stage) (Table 1). Seven normal donor corneas (age range 53 to 83 years) were obtained from the Lions NSW Eye Bank with consent and ethics approval (Table 2).

**Table 1:** Characteristics of KC Patients.

aKC	Gender	Age at diagnosis (yrs)	Age at surgery (yrs)	Contact Lenses (Y or N)	History Allergy/atopy (Y or N)	bDALK (Y or N)
1	F	24	32	Y	N	N
2	F	23	30	N	Y (asthma)	Y
3	F	30	37	N	N	Y
4	M	27	32	N	Y (atopy/asthma)	N
5	M	25	32	N	N	Y
6	F	18	21	N	Y (atopy/asthma)	N
7	M	24	28	Y	N	N
8	M	24	43	Y	N	N
9	F	20	31	Y	N	N
10	F	32	38	Y	N	Y
11	M	22	37	Y	N	Y
12	F	21	31	N	Y (atopy)	Y
13	M	18	75	N	N	N
c14	M	55	63	N	N	Y
15	F	24	28	Y	N	Y

a Grade 4 KC: Severe; VA >6/7.5 with contact lens correction; severe corneal thinning and Munson’s sign.

b DALK: Deep anterior lamellar keratoplasty.

c Ectasia developed post-LASIK.

**Table 2:** Characteristics of Control Corneas.

Control	Gender	Age (yrs)
1	M	67
2	F	53
3	F	64
4	F	67
5	M	83
6	M	63
7	M	83

KC corneal buttons (∼8 mm diameter), with the cone marked indicating its central location, were fixed in 10% neutral buffered formalin (NBF); whole corneas were fixed in 2% paraformaldehyde/phosphate buffered saline (PBS) (pH 7.4). All specimens were subsequently paraffin embedded, and sections cut at 6 µm and collected on Super-Frost Plus slides (Menzel-Glaser, Saarbruckener, Germany).

### Histopathology

Sections were dewaxed in xylenes, rehydrated through a series of alcohols to water and stained with Mayer's hematoxylin and eosin, dehydrated through a series of alcohols and xylenes, mounted in DPX (Sigma-Aldrich, St. Louis, MO, USA) and coverslipped. A separate series of sections stained with Periodic Acid Schiff (PAS) using standard protocols were also examined. Briefly, sections were dewaxed and rehydrated prior to incubation in 0.5% periodic acid solution, followed by staining in Schiff's reagent (HD Scientific Supplies, Wetherill Park, NSW, Australia). After further rinsing in water, sections were stained with Mayer’s haematoxylin, rinsed in water, then dehydrated through a series of alcohols and xylenes, and mounted in DPX and coverslipped. Sections were examined with an Olympus DP70 light microscope (Olympus, Center Valley, PA, USA).

**Figure 1:**
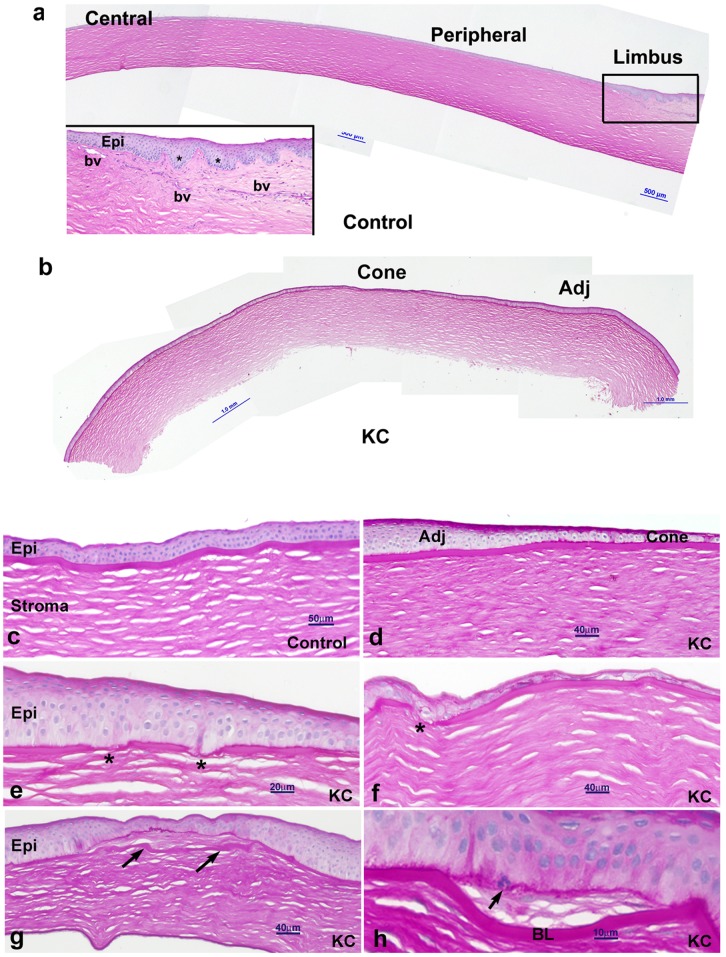
Light microscopy of control and KC corneas stained with PAS and Mayer's hematoxylin. **a.** Control corneas showed uniform thickness multilayered epithelium in the central region, with increased stromal thickness towards the limbus. Blood vessels and undulating basal epithelium (asterisks) are only seen in the limbus region. **b.** In KC buttons, obvious epithelial thinning associated with stromal thinning is seen more centrally. **c to h.** Representative images at higher magnification show (c) control corneal epithelium and anterior stroma, (d) obvious epithelial thinning in the KC cone compared to the adjacent epithelium. (e, f) Enlarged basal epithelial cells and irregular Bowman’s layer (thinning and breaks, asterisks) are common features in KC specimens. In some KC specimens (g) evidence of incursion of stromal tissue between Bowman’s layer and the epithelium is seen (arrows). (h) A leukocyte is also seen in the KC basal epithelium, where stroma invaded between epithelium and Bowman’s layer (arrows). Epi: epithelium; bv: blood vessel; Adj: adjacent region; BL: Bowman’s layer.

Two regions were identified in sections of KC buttons: a more central thinner cone region with a transition to an adjacent region with a thicker epithelium. In control corneas, central, peripheral and limbal regions were identified (Fig. 1a, b). These regions were used for semi-quantitative grading of immunostained sections as described below.

### Immunohistochemistry

Sections were dewaxed and rehydrated through alcohols to water. For antigen retrieval, sections were incubated in 0.01 M citrate buffer (pH 6) at 85°C for 10 minutes, cooled to 40°C, and rinsed in Tris-buffered saline (TBS, pH 7.4) with 0.1% Tween-20 (TBST). Sections were incubated at room temperature (RT) in 5% bovine serum albumin (BSA) in TBST for 30 minutes, followed by incubation overnight at 4°C in appropriate primary antibodies or negative controls (Table 3). After overnight incubation, sections were washed in TBST, and incubated in either donkey anti-rabbit Alexa 488 (2 µg/ml, Molecular Probes, Life Technologies, New York, USA) or donkey anti-goat Alexa 488 (2 µg/ml, Molecular Probes) for 2 h at RT, and washed in TBST. Negative controls were treated identically, using non-specific immunoglobulins (Ig) (rabbit or goat) at the same concentration as for primary antibodies (Table 3). Cell nuclei were counter-stained with propidium iodide (PI) (0.1 µg/ml, Molecular Probes) for 5 min, followed by rinsing in TBST. Immunolabelling was repeated at least twice per specimen for each antibody, and appropriate Ig controls were included for each experiment. Slides were mounted in 20% glycerol/PBS, coverslipped, sealed with nail varnish, and viewed using the Zeiss LSM700 scanning laser confocal microscope and image software (Zen 2011, Carl Zeiss MicroImaging GHBH, Jena, Germany). Multichannel excitation bleedthrough was minimized by using fluorochromes with a large difference in peak excitation. Emission bleedthrough was minimized by multitracking, where signal crosstalk between neighbouring channels was corrected by performing a sequential image capture routine.

**Table 3:** Antibodies used for Immunohistochemistry.

Protein	Species	Antibody/Company	Concentration
SFRP1	rabbit	sc-13939/Santa Cruz Biotechnology, Santa Cruz, CA, USA	4 µg/ml
SFRP2	rabbit	GTX111892GeneTex, Irvine, CA, USA	3.8 µg/ml
SFRP3	goat	AF192/R&D Systems, Minneapolis, MN, USA	1 µg/ml
SFRP4	rabbit	sc-30152/Santa Cruz	4 µg/ml
SFRP5	goat	sc-14331/Santa Cruz	4 µg/ml
Rabbit Ig	rabbit	Jackson ImmunoResearch Laboratory, West Grove, PA, USA	3.8 or 4 µg/ml
Goat Ig	goat	Jackson ImmunoResearch Lab.	1 or 4 µg/ml

We also assessed antibody specificity using immunoblotting and recombinant proteins or blocking peptides as positive controls, and used antigen pre-absorption to assess antibody specificity on sections (not shown).

### Semi-quantitative Analysis

A semi-quantitative grading scale was used to assess the intensity and distribution of SFRP immunoreactivity of the epithelium, stroma and endothelium. Grading for KC buttons was made in the region adjacent to the cone (Adj), and for control corneas, in a similar central corneal region (Fig. 1a, b). Immunolabelling of the thinned cone area of KC buttons was examined in each sample but was not graded. The grading scale was based on the intensity of the immunofluorescence graded visually (0 = no staining, 0.5 = very weak, 1 = weak, 2 = moderate and 3 = strong), and the percentage (%) of immunolabelling (0 = 0%, 1 = 1% to 10%; 2 = 11% to 50% and 3>50%). A final grade of 0 to 6 (intensity+% immunolabelled) was then given for each specimen.

### Statistical Analysis

Semi-quantitative grades were presented as mean ± SEM, and a Mann-Whitney test was used for comparison of SFRP immunolabelling of KC and control corneas. A p value of <0.05 was considered statistically significant. Statistical analysis was performed using GraphPad Prism 5 software (GraphPad Software, CA, USA).

## Results

### Histopathology

Control specimens comprised central and peripheral cornea and a limbal region (Figure 1a). The central control cornea had five to eight layers of epithelial cells, stroma, and intact Descement’s membrane and endothelium; more peripherally the epithelium and especially stroma increased in thickness (Figure 1a, c). In the limbal region, blood vessels were visible within the more anterior limbal stroma, just below the undulating limbal epithelium (Figure 1a, see inset). PAS-positive goblet cells were sometimes seen within the conjunctival epithelium (not shown).

KC buttons showed irregular and thinned layers of epithelium (one or two layers) within the cone region, with a gradual transition to adjacent epithelium comprising five to eight layers of epithelial cells (Figure 1b, d and e). In KC, epithelial cells showed localised detachments from the underlying basement membrane, with focal thickening of the epithelial basement membrane (Figure 1h). Disruption and breaks in Bowman’s layer, and anterior stromal irregularities with associated stromal thinning were also seen within the cone region and in adjacent regions of KC buttons (Figure 1d to h). Approximately 50% of KC buttons (8/15) were surgically removed using deep anterior lamellar keratoplasty (DALK) and often displayed stromal artefacts and no endothelium (Figure 1b).

### Immunohistochemistry

**Figure 2:**
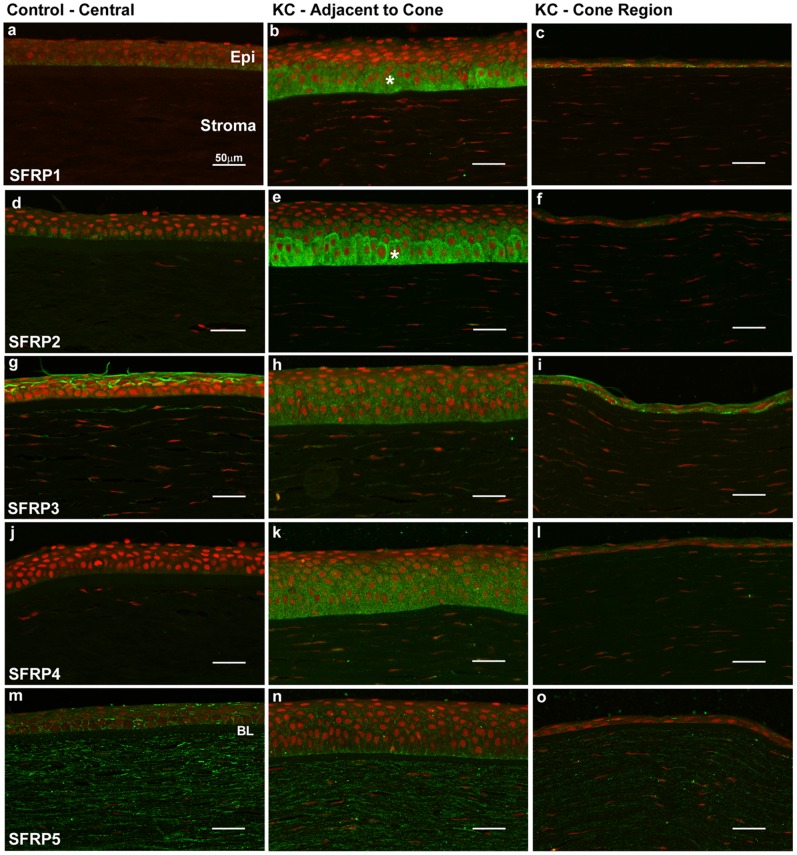
Representative confocal microscope images showing immunolabelling for SFRP1 to 5 in central control cornea and KC buttons. Increased cytoplasmic SFRP1 to SFRP4 immunoreactivity was detected in the basal epithelium adjacent to the cone region of KC, when compared to controls. **a to c:** Increased cytoplasmic SFRP1 staining was detected in the basal epithelial layer in the adjacent to cone region in KC cornea (asterisks, b) compared to the cone region (c) and control cornea (a). **d to f:** Stronger SFRP2 staining was also detected in the basal epithelial layer in the adjacent to cone region in KC cornea (asterisks, e) compared to the cone region (f) and control cornea (d). Weak SFRP2 staining was detected in the stroma in KC and control cornea (d-f). **g to i:** Strong SFRP3 cell membrane staining was observed in the superficial epithelial layers in control cornea, weak SFRP3 cytoplasmic staining was observed (g). KC cornea showed strong cytoplasmic SFRP3 immunostaining in both adjacent to cone epithelium (h) and cone region (i). SFRP3 was also detected in the keratocytes of in control and KC cornea. **j to l:** Strong epithelial cytoplasmic SFRP4 immunostaining was seen in KC, most obviously in the epithelium adjacent to the cone region, compared to controls where no SFRP4 was detected. No SFRP4 was observed in keratocytes. **m to o:** Epithelial cell membrane and strong stromal SFRP5 expression was observed in both control (m) and KC specimens (n-o). Scale bar = 50 µm.

**Figure 3:**
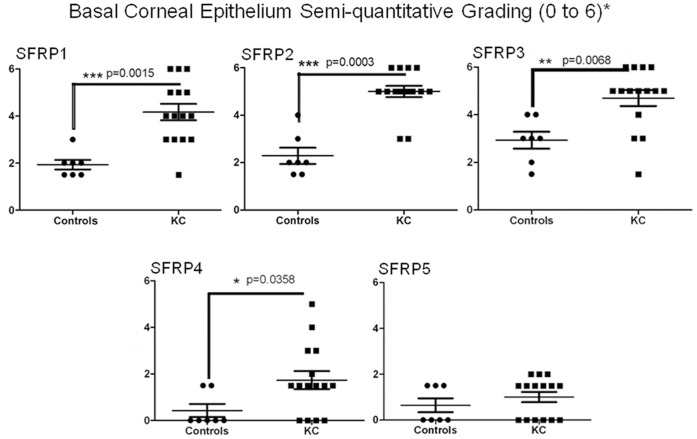
Graphs summarising semi-quantitative grading for SFRP immunolabelling of KC and control specimens. The graphs show the range of grading for SFRP1 to 5 immunostaining in the basal epithelium adjacent to the cone region, and a similar region in control corneas. SFRP1 to 4 immunostaining is significantly increased compared to controls (p<0.05, Mann-Whitney test).

In KC buttons, moderate to strong cytoplasmic immunostaining for SFRP1 to 4 was seen within the basal and wing cell epithelium adjacent to the cone region, compared to no or weak immunostaining in similar regions within control corneas (Figure 2, Figure S1). SFRP3 and to a lesser extent, SFRP5, also showed epithelial cell membrane immunolabelling of both KC and control specimens (Figure 2, Figure S1). Irregular flattened (squamous) epithelial cells within the cone region of KC buttons showed weak, more diffuse cytoplasmic SFRP immunostaining, consistent with the much reduced cytoplasm of these cells (Figure 2, Figure S1). Semi-quantitative analysis showed significantly increased immunolabelling for SFRP1 to 4, but no SFRP5, within the KC basal epithelium compared to control corneas at similar locations (Figure 3; Table 4).

**Table 4:** SFRP Immunolabelling: Semi-Quantitative Grading^a.^

SFRP Protein	Basal epithelium (mean ± SEM)		Sig^b^	Epithelial cell membrane (mean ± SEM)		Sig	cStroma (mean ± SEM)		Sig	Endothelium (mean ± SEM)		Sig
	Control	KC	dp	Control	KC	p	Control	KC	p	Control	fKC	p
SFRP1	1.6±0.3	4.2±0.4	**0.015**	ND^e^	ND	–	0.7±0.5	0.5±0.2	NS	3.9±0.2	4.1±0.3	NS
SFRP2	2.3±0.3	5.0±0.2	**0.0003**	ND	ND	–	0.6±0.4	0.8±0.2	NS	3.2±0.7	1.9±1.1	NS
SFRP3	2.9±0.4	4.7±0.3	**0.0068**	4.3±0.7	5.0±0.4	NS	1.9±0.5	2.6+0.2	NS	4.3±0.7	5.0±0.4	NS
SFRP4	0.4±0.3	1.7±0.4	**0.0358**	ND	ND	–	ND	ND	–	ND	ND	–
SFRP5	0.6±0.3	1.0±0.2	NS	2.8+0.5	1.6+0.3	NS	4.7+0.2	5.0+0.1	NS	ND	ND	–

a Semi-quantitative grading = Intensity+% immunolabelling (minimum = 0 and maximum = 6):

*Intensity of Immunoreactivity.*

0 = no staining.

0.5 = very weak.

1 = weak.

2 = moderate.

3 = strong.

*% Immunolabelling.*

0 = 0%.

1 = 1% to 10%.

2 = 11% to 50%.

3 = >50%.

b Significance.

c SFRP1, 2 and 3 expressed in keratocytes only; SFRP5 expressed in stroma including matrix and cells.

d Mann-Whitney test, p<0.05 significant; NS - not significant.

e ND - not detected.

f KC full thickness graft n = 7/15; deep anterior lamellar keratoplasty (DALK) n = 8/15.

#### SFRP1

Moderate to strong heterogeneous cytoplasmic SFRP1 expression was seen in basal and wing cell epithelial layers in all KC specimens particularly in the region adjacent to the cone in KC specimens, compared to similar regions in control corneas, where SFRP1 immunolabelling was weak or not apparent (Figure 2a and b). SFRP1 expression in the KC corneal epithelium adjacent to the cone was consistently graded significantly higher than similar regions of epithelium in control corneas (Figure 3, p<0.0015; Table 4). Keratocytes, stroma and endothelium of both KC and control specimens showed no or weak expression of SFRP1 across the whole specimen, and this was not significantly different between the two groups (Table 4; Figure S1a).

#### SFRP2

SFRP2 expression in KC buttons was strongest of the SFRP family proteins investigated (Figure 2; Table 4), with a similar expression pattern to SFRP1 in KC. Basal/wing epithelial cells showed strong cytoplasmic SFRP2 immunoreactivity in KC corneas, compared to weak SFRP2 expression in control central corneal epithelium. This was significantly increased in KC compared to similar regions in control corneas (Figure 3, p<0.0003; Table 4). Keratocytes, stroma and endothelium of both KC and control corneas showed similar expression of SFRP2 that was not significantly different between the two groups (Table 4; Figure S1b).

#### SFRP3

SFRP3 immunostaining of epithelial cell membranes was seen in both KC and controls across the whole specimen (Figure S1c). Similar to SFRP1 and 2, significantly increased cytoplasmic SFRP3 was observed in KC basal/wing cell epithelium compared to controls (Figure 2g and h; Figure 3, p<0.0068; Table 4). In the KC cone region, epithelial cells also displayed cell membrane and cytoplasmic staining (Figure 2i). Stromal keratocytes and endothelium showed low to moderate SFRP3 that was similar in both KC and control corneas (Table 4, Figure S1c).

#### SFRP4

**Figure 4:**
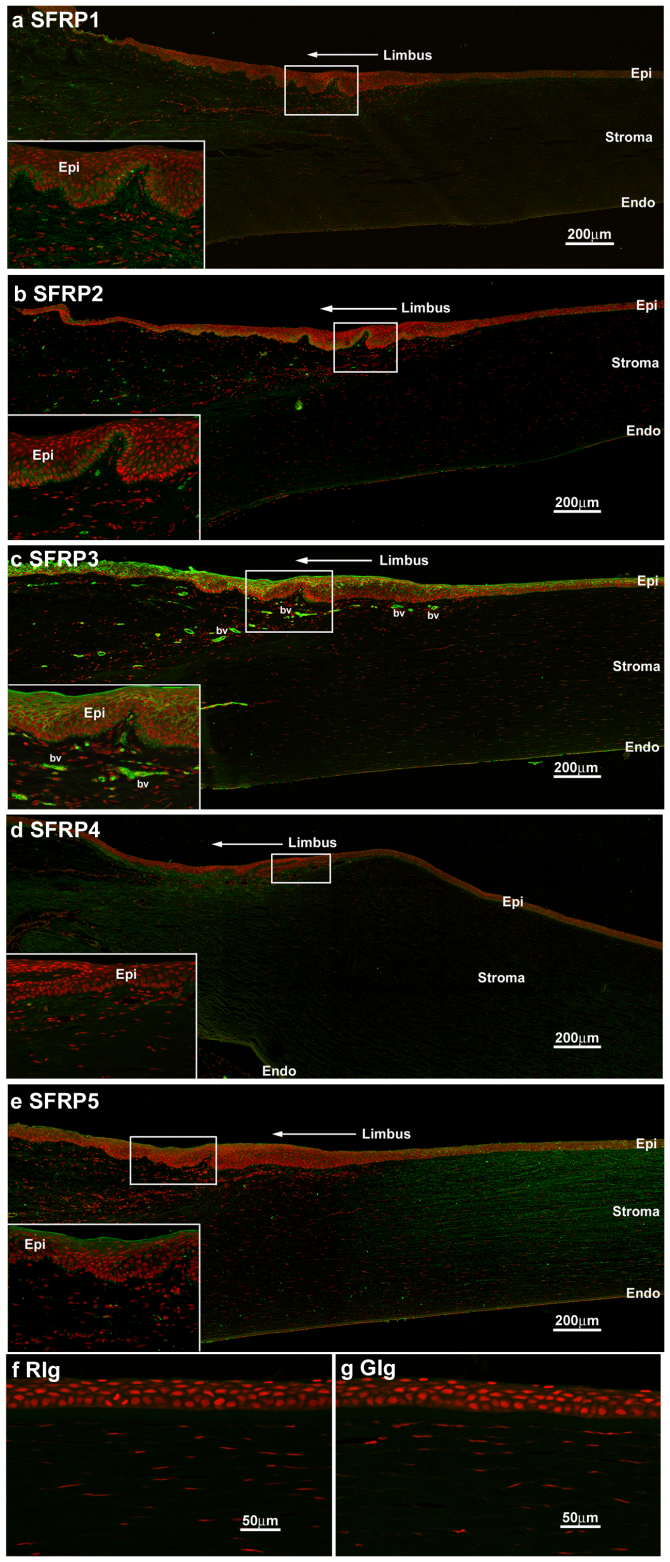
SFRP1 to 5 immunostaining in the control cornea limbus and periphery, and Ig controls. **a.** Basal epithelium and stroma of the limbus showed moderate SFRP1 staining. **b.** Moderate SFRP2 staining was detected in the basal epithelium only. **c.** Strong SFRP3 epithelial cell membrane staining was found in the superficial epithelial layers in the limbus. Blood vessels (bv) in the limbal region also stained strongly for SFRP3. **d.** SFRP4 staining could not be detected in the limbus epithelium, although moderate SFRP4 staining was seen in limbal stroma. **e.** Strong SFRP5 immunoreactivity was found in the superficial epithelial layers in the limbus. Compared to more central cornea, limbal and peripheral corneal stroma showed weaker SFRP5 immunostaining. **f and g.** No immunostaining was detected in KC or control corneas for (f) rabbit Ig or (g) goat Ig negative controls included in each experiment. Epi: epithelium; Endo: endothelium; bv: blood vessel.

Cytoplasmic SFRP4 expression was seen in KC basal epithelium (Figure 2k and l), and this was significantly increased compared to control corneas (Figure 2j; Figure 3, p<0.0358; Table 4). In control corneas, SFRP4 was not expressed centrally but was apparent at the low levels in the basal epithelium near the limbus (Figure 4). SFRP4 was not observed in keratocytes, stroma or endothelium of either control or KC buttons (Table 4; Figure S1d).

#### SFRP5

Weak SFRP5 cell membrane labelling was seen for both control and KC corneal epithelium (Figure 2m to o; Table 4). However, SFRP5 was strongly expressed within the stroma of both control and KC corneas, but not in the endothelium (Table 4; Figure S1e).

#### Expression of SFRPs in control peripheral cornea and limbus

SFRP1, 2 and 4 showed weak immunolabelling of the limbal epithelium, mostly in the basal epithelial layers. SFRP1 and 4 were also detected within the limbal stroma, in contrast to the more central corneal stroma. Reduced SFRP5 immunoactivity was observed in the limbal stroma compared to the central corneal stroma (Figure 4).

Peripheral corneal, limbal and conjunctival epithelium showed obvious cell membrane immunoreactivity for SFRP3 and 5, that was however reduced compared to the central corneal epithelium (Figure 4c, e). SFRP5 immunostaining was obviously less in the limbal stroma compared to the strong expression seen in the corneal stroma (Figure 4e). Limbal stromal blood vessels displayed weak to moderate SFRP2 and strong SFRP3 immunolabelling (Figure 4b, c).

#### Negative controls

For each experiment, rabbit or goat immunoglobulins (Ig) were included as negative controls in parallel with SFRP antibodies. No evidence of non-specific immunolabelling for either goat or rabbit Ig was seen in KC or control specimens (Figure 4f, g).

## Discussion

We examined the expression and distribution of SFRP1 to 5 in control corneas and KC buttons. Control corneas expressed low to moderate levels of these proteins however KC buttons showed increased expression and altered distribution of SFRPs. Specifically, significantly higher levels of SFRP1 to 4 are detected in the KC basal corneal epithelium adjacent to the cone region, compared to similar regions in control corneas.

### The Potential Role of SFRPs in KC

Currently little is known about the expression and function of SFRPs in normal or pathological human corneas. We observed significantly upregulated expression of SFRPs in KC compared to control corneas, suggesting involvement of SFRPs in the pathogenesis of KC. Corneal studies have found that exogenous SFRP1 can block Wnt signalling, and may inhibit Wnt-7a promoted cell proliferation, delaying corneal wound healing [17]. More recently, Zhou et al (2011) showed that exogenous SFRP1 can prevent corneal epithelium cell death induced by benzalkonium chloride (a preservative know to be toxic to corneal epithelium), which may act via the Wnt signalling pathway [18]. These findings suggest that different Wnt signalling pathways may affect corneal epithelial cell apoptosis and proliferation depending on the conditions.

The Wnt canonical pathway has been reported to regulate the proliferation of adult human corneal limbal stem cells [16]. However, this study primarily investigated the expression of Wnt molecules, and mRNA for only SFRP3 and 5 [16]. Whether SFRPs detected in KC are associated with Wnt signalling pathways remains to be established. In preliminary studies, we found that β-catenin and LEF1 (key molecules in canonical Wnt pathways [20]) showed more cytoplasmic staining in KC than control corneas, with no obvious nuclear translocation, suggesting that the canonical pathway may be unaffected in KC (data not shown). Interestingly, in another degenerative eye disease, retinitis pigmentosa (RP), immunostaining for SFRP2 and β-catenin showed up-regulation of SFRP2, but no clear β-catenin differences [21]. In this context, it is unclear how SFRP2 interacts in the Wnt signalling pathway, although the authors speculated it could be associated with cell apoptosis in a complicated network [21]. Further investigations of both canonical and non-canonical Wnt signalling pathways in the human cornea are needed.

SFRPs have been widely studied in other biological systems, particularly in cancer. Down regulation of SFRP1, SFRP4 and 5 are reported in aggressive breast cancer [22], ovarian cancer [23] and gastric cancer [24] respectively. Overexpression of SFRP1 and 2 can inhibit cell growth, transformation and invasion in cervical cancer [25], and increase the invasiveness of renal cancer cells [26]. Overexpression of SFRP1 and SFRP3 has been detected in advanced renal cancer cells [26], [27]. Up-regulation of SFRP1 has also been reported to induce apoptosis in osteoblast cells [28]. Conversely, dental studies showed that SFRP1 expression can be induced by apoptosis caused from ceramide [29] or *P. gingivalis* [30]. These findings are consistent with SFRPs affecting a wide range of cellular activities differently depending on the conditions [31], and indicate they are important related to cell apoptosis and migration. The other question is whether the expression of SFRPs is specific for KC-associated corneal degeneration. We observed that corneal epithelium in KC showed increased SFRP1 to 4 compared to control corneas. However, when we examined a small series of bullous keratopathy corneas (n = 3, Figure S2), no or weak expression of SFRPs was observed in the corneal epithelium. Iqbal et al., (2013) also noted that compared to KC, Fuch's dystrophy corneas showed low level expression of SFRP1. KC is primarily an anterior degenerative corneal condition involving epithelial disorganisation and anterior stromal degeneration [32]. In contrast, bullous keratopathy typically develops following corneal endothelial damage (for example, associated with Fuch's dytrophy or post-intraocular lens surgery), leading to stromal and epithelial oedema, with secondary loss of epithelial attachment to the underlying Bowman’s layer and subepithelial fibrosis [33]. Although further studies are needed, these observations suggest that upregulated SFRP expression in basal corneal epithelium is KC-specific.

We also noted that cytoplasmic expression of SFRPs varied significantly in KC buttons compared to control corneas. This contrasts with no obvious differences between KC and controls for SFRP3 and 5 cell membrane immunostaining, and extracellular matrix SFRP5 expression. Although it is unclear how the altered cellular location of SFRPs may be associated with KC, studies in other tissues suggest that the location of SFRP proteins could be functionally important. For example, cell membrane SFRP4 is reported to correlate with a good prognosis in prostate cancer [34], suggesting that functional cell membrane SFRP4 bound to Wnt ligands to inhibit activation of the Wnt signalling pathway in prostate cancer [34]. Furthermore, a strong association of SFRP5 and extracellular matrix has been reported in adipose tissue [35]. SFRP5 was reported to be tightly bound to the extracellular matrix, suppressing oxidative metabolism; bound SFRP5 could only be released using heparin [35]. Finally, the overexpression of SFRPs in KC may be a consequence of epithelial damage or in response to underlying anterior stromal degeneration, including apoptotic death of anterior stromal keratocytes as reported by Kim et al. [7]. The significance of these observations for KC pathogenesis and corneal function remain to be explored.

### Does KC Affect more than the Cone Region?

KC is generally considered to initially affect the more anterior cornea, with the posterior cornea, including Descemet’s membrane and endothelium, remaining unaffected until more advanced stages of the disease (Grade 4). The pathology is characterised by a progressive thinning of the stroma and epithelium that leads to corneal ectasia, clinically described as the “cone”. In our study, all KC buttons showed histopathology consistent with the many other studies in KC [36]–[38], with a more central region of epithelial thinning, disruption of the epithelial basement membrane, breaks in Bowman’s layer and anterior stromal thinning. Previous studies have suggested that in KC, not only the cone region, but also the mid-peripheral cornea is affected. Mathew et al. (2011) observed a significantly larger area of abnormal Bowman’s layer in KC (>50% of the assessed area and wider than the cone region), which could not be simply explained by localised abnormalities in the cone region [36]. Brautaset et al. (2012) measured corneal thickness in KC using Visante optical coherence tomography and Orbscan II, and reported significant thinning in more peripheral cornea compared to controls, consistent with more than the cone region being affected in KC. We detected significantly higher expression of SFRPs across the whole KC button, indicating that the pericentral corneal epithelium is also affected in KC. This observation could be clinically important in further understanding the underlying cause of recurrence of KC seen in some patients following penetrating keratoplasty. It has been suggested that the host tissue is affected in KC recurrence, and may play a role in this process [39]; conversely, another study suggested that the donor cornea may be affected by very early stage (undetected) KC [40]. Our observations favour the former theory, that the remaining host tissue in KC patients may be affected.

### The Role of Basal Epithelium in Epithelium Homeostasis and KC Development

In advanced KC, columnar basal epithelial cells are not seen within the cone region, with just one or two layers of squamous epithelium observed. However, histopathology and immunolabelling of the epithelium surrounding the cone region, showed enlarged columnar basal epithelial cells compared to control cornea. In addition, cytoplasmic overexpression of SFRP1 to 4 was observed mainly in the basal epithelial cells adjacent to the cone region of KC buttons. The abnormal morphology and SFRP expression in the KC basal epithelial cells suggest that these cells are affected in KC, and may be a major site for the aberrant cellular activities.

Since basal epithelial cells are important in maintaining the uniform thickness of the cornea and epithelial basement membrane, impaired epithelial maintenance may be a feature of KC pathogenesis, leading to chronic, epithelial thinning. An *in vitro* study has shown that corneal epithelial cells secrete basal lamina and native striated collagen fibrils important for formation of the basement membrane [41], [42]. Compromise of the epithelial basement membrane, characteristically seen in KC, may thus be a consequence of impaired epithelial maintenance.

As noted above, the epithelium is critical in maintaining normal corneal function and integrity, and the basal epithelium of the central cornea and limbus is considered crucial in the most widely accepted theory of corneal epithelium homeostasis – the corneal limbal stem cell theory [43]. Briefly, corneal limbal stem cells (LSCs) residing in the basal limbal region are proposed to proliferate and differentiate to transient amplifying cells (TACs), which move towards the central basal epithelium, continually proliferate to maintain the basal epithelial layer, and then migrate upwards and differentiate to form wing cells. Wing cells will continue migrating upwards to the superficial layer to replace superficial cells lost from the corneal surface [43].

More recently another theory - the corneal epithelial stem cell (CESC) hypothesis – has been proposed to explain ongoing renewal of corneal epithelium [44]. This was first suggested by Majo et al., (2008), who showed that mouse corneal epithelium is self-maintained and contained oligopotent stem cells in the basal epithelium capable of generating mucin-producing goblet cells. For porcine cornea, they found the entire ocular surface contained oligopotent stem cells capable of generating both corneal and conjunctival cells [45]. More recently, it has been proposed that both theories (LSC and CESC) are complementary, CESCs maintaining the corneal epithelium during normal homeostasis, with LSCs being more important during wound healing [44].

### Differential Expression of SFRPs in the Control Cornea versus Limbus

Differential expression was observed in control cornea and limbus for all SFRPs. These different staining patterns most likely reflect differences in the structure and microenvironment within these regions. Morphologically the limbus is different to central cornea. The epithelium becomes undulating associated with the papillae of the Palisades of Vogt, the niche regions, which are generally considered responsible for corneal epithelial repopulation (see however previous section discussing both LSCs and CESCs). Both intrinsic factors and the surrounding niche microenvironment are critical for maintaining stem cell characteristics and cell differentiation [2], [46]. Activation of the canonical Wnt signalling pathway by LiCl has been shown to increase LSC proliferation *in vitro*, and increased SFRP3 and 5 mRNA expression have been detected in the limbus compared to central cornea, although protein expression was not investigated [16]. To date, protein studies of limbal SFRPs have not been performed [16]. Our findings suggest that the expression of SFRPs in normal human cornea is tightly controlled in different corneal regions, including the limbus; whether this is associated with the Wnt signalling pathway remains to be determined.

The central cornea is avascular, however blood vessels are normally found within the conjunctiva and anterior limbal stroma. We observed that the limbal vessels immunolabelled strongly for SFRP3 (and to a lesser extent, SFRP2), suggesting potential for SFRP3 as a vascular marker. Recent studies using angiogenesis assays in a renal cancer model showed that SFRP3 can enhance the formation of capillary-like tubular structures, via the Tie2/angiopoietin system [27]. Further investigation of the role(s) of SFRP3 (and SFRP2) in blood vessel growth are required.

### Conclusion

We observed significantly increased expression of SFRPs in KC compared to control corneas; specifically the epithelium adjacent to the cone region showed obvious SFRP1 to 4 immunostaining consistent with involvement of the pericentral corneal epithelium in KC. Differential expression of SFRPs in control central cornea and limbus is consistent with differences in the microenvironment of these regions. Taken together, our results further support a role for SFRPs in maintaining a healthy cornea and in the pathogenesis of KC.

## Supporting Information

Figure S1 **Low power images of SFRP1 to 5 immunostaining in KC and control corneas.** **a.** In KC, obvious SFRP1 immunostaining is detected in the epithelium adjacent to cone region. Control corneas showed weak immunostaining for SFRP1. SFRP1 was also detected in the endothelium of both KC and control cornea. **b.** Strong SFRP2 immunostaining was detected in the epithelium adjacent to cone, compared to the cone region in KC and control cornea. SFRP2 was also detected in the endothelium of both KC and control cornea. **c.** Strong epithelial cell membrane staining for SFRP3 was found in controls. In KC, we noted strong cytoplasmic immunostaining for SFRP3. Both KC and control cornea showed stromal and endothelial SFRP3 expression. **d.** Obvious SFRP4 immunostaining is found in the KC epithelium adjacent to the cone region, compared to no or weak expression in the cone region and control corneal epithelium. SFRP4 immunostaining were found in the stroma and endothelium of KC and control corneas. **e.** Both KC and control specimens showed strong stromal expression for SFRP5. Cytoplasmic SFRP5 was also seen in the KC epithelium adjacent to cone, but not in the control corneas.
(JPG)

Figure S2 **SFRP1 to 5 immunostaining in bullous keratopathy. a.** Weak SFRP1 immunostaining was seen in remnants of epithelium in bullous keratopathy. SFRP1 was not apparent in the stroma or endothelium (not shown). **b.** SFRP2 immunostaining was not detected in the epithelium, stroma or endothelium (not shown) in bullous keratopathy. **c.** Strong epithelial cell membrane staining for SFRP3 was found in bullous keratopathy; an area of subepithelial fibrosis is also seen (**). **d.** Obvious SFRP4 immunostaining is seen associated with subepithelial fibrosis (**). SFRP4 immunostaining however was not detected in epithelium, stroma or endothelium (not shown). **e.** No SFRP5 was detected in the epithelium (not shown), stroma or endothelium in bullous keratopathy.
(JPG)
