# Supplementary figures and images for: Expression of SFRP Family Proteins in Human Keratoconus Corneas
Source: PLoS One. 2013 Jun 18;8(6):e66770. doi: 10.1371/journal.pone.0066770 (PMC3688946; doi:10.1371/journal.pone.0066770)

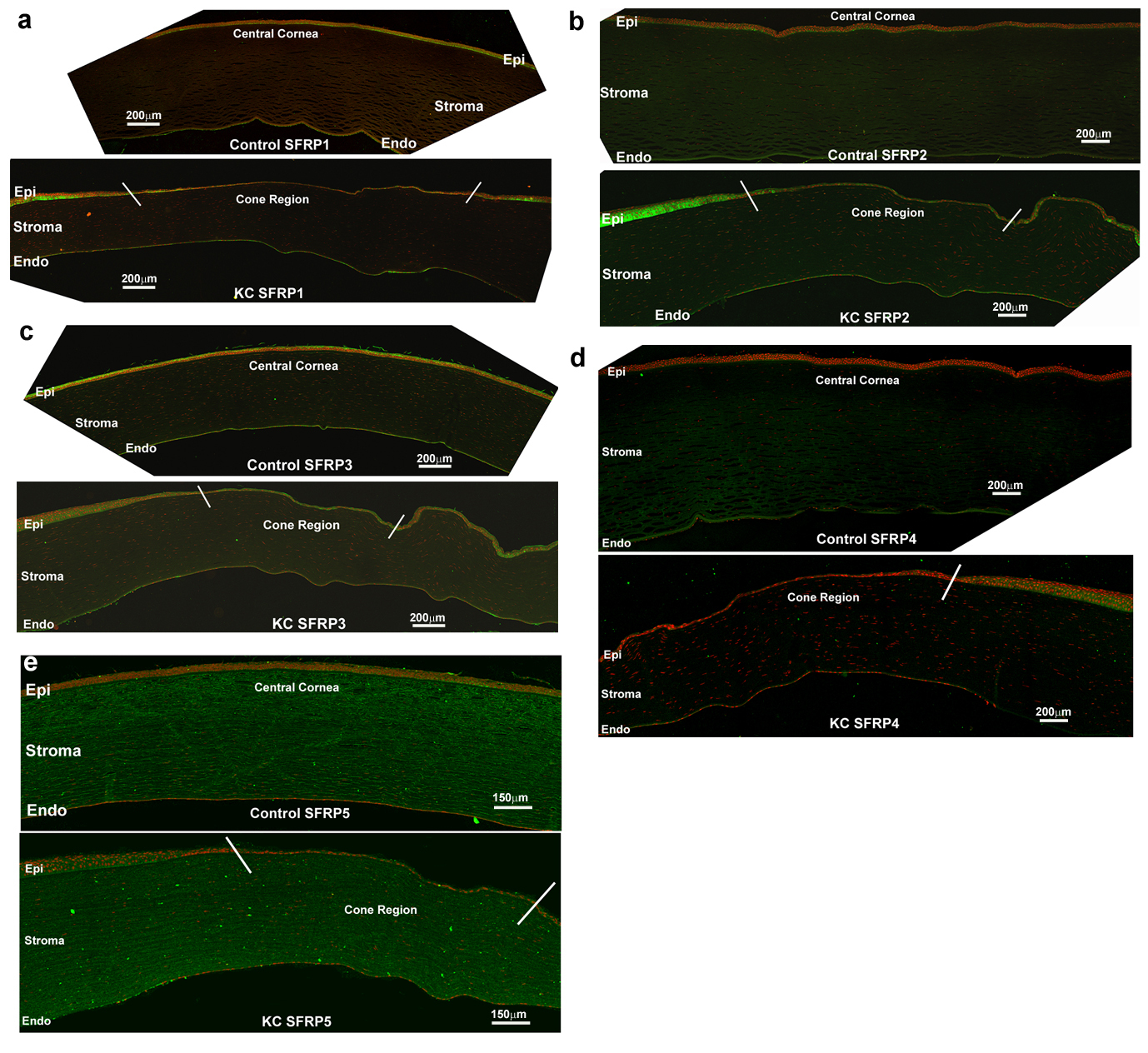

Supplement: Figure S1 — Low power images of SFRP1 to 5 immunostaining in KC and control corneas. a. In KC, obvious SFRP1 immunostaining is detected in the epithelium adjacent to cone region. Control corneas showed weak immunostaining for SFRP1. SFRP1 was also detected in the endothelium of both KC and control cornea. b. Strong SFRP2 immunostaining was detected in the epithelium adjacent to cone, compared to the cone region in KC and control cornea. SFRP2 was also detected in the endothelium of both KC and control cornea. c. Strong epithelial cell membrane staining for SFRP3 was found in controls. In KC, we noted strong cytoplasmic immunostaining for SFRP3. Both KC and control cornea showed stromal and endothelial SFRP3 expression. d. Obvious SFRP4 immunostaining is found in the KC epithelium adjacent to the cone region, compared to no or weak expression in the cone region and control corneal epithelium. SFRP4 immunostaining were found in the stroma and endothelium of KC and control corneas. e. Both KC and control specimens showed strong stromal expression for SFRP5. Cytoplasmic SFRP5 was also seen in the KC epithelium adjacent to cone, but not in the control corneas. (JPG) [file pone.0066770.s001.jpg]

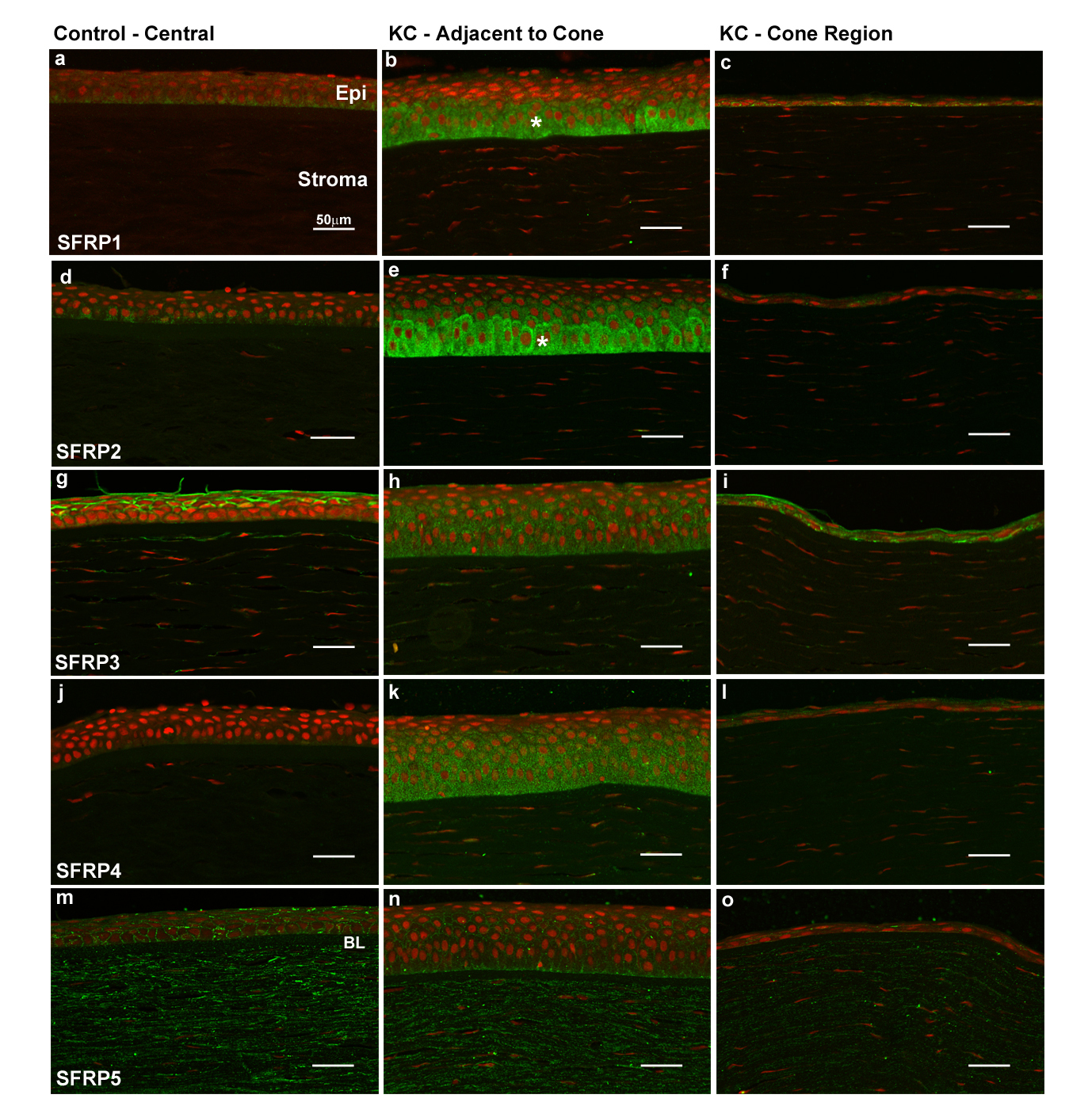

Supplement: Figure S2 — SFRP1 to 5 immunostaining in bullous keratopathy. a. Weak SFRP1 immunostaining was seen in remnants of epithelium in bullous keratopathy. SFRP1 was not apparent in the stroma or endothelium (not shown). b. SFRP2 immunostaining was not detected in the epithelium, stroma or endothelium (not shown) in bullous keratopathy. c. Strong epithelial cell membrane staining for SFRP3 was found in bullous keratopathy; an area of subepithelial fibrosis is also seen (**). d. Obvious SFRP4 immunostaining is seen associated with subepithelial fibrosis (**). SFRP4 immunostaining however was not detected in epithelium, stroma or endothelium (not shown). e. No SFRP5 was detected in the epithelium (not shown), stroma or endothelium in bullous keratopathy. (JPG) [file pone.0066770.s002.jpg]
